# Predicting histologic grades for pancreatic neuroendocrine tumors by radiologic image-based artificial intelligence: a systematic review and meta-analysis

**DOI:** 10.3389/fonc.2024.1332387

**Published:** 2024-04-23

**Authors:** Qian Yan, Yubin Chen, Chunsheng Liu, Hexian Shi, Mingqian Han, Zelong Wu, Shanzhou Huang, Chuanzhao Zhang, Baohua Hou

**Affiliations:** ^1^Department of General Surgery, Guangdong Provincial People’s Hospital (Guangdong Academy of Medical Sciences), Southern Medical University, Guangzhou, China; ^2^School of Medicine, South China University of Technology, Guangzhou, China; ^3^Department of General Surgery, Heyuan People’s Hospital, Heyuan, China

**Keywords:** pancreatic neuroendocrine tumors, meta-analysis, radiomics, machine learning, deep learning

## Abstract

**Background:**

Accurate detection of the histological grade of pancreatic neuroendocrine tumors (PNETs) is important for patients’ prognoses and treatment. Here, we investigated the performance of radiological image-based artificial intelligence (AI) models in predicting histological grades using meta-analysis.

**Method:**

A systematic literature search was performed for studies published before September 2023. Study characteristics and diagnostic measures were extracted. Estimates were pooled using random-effects meta-analysis. Evaluation of risk of bias was performed by the QUADAS-2 tool.

**Results:**

A total of 26 studies were included, 20 of which met the meta-analysis criteria. We found that the AI-based models had high area under the curve (AUC) values and showed moderate predictive value. The pooled distinguishing abilities between different grades of PNETs were 0.89 [0.84-0.90]. By performing subgroup analysis, we found that the radiomics feature-only models had a predictive value of 0.90 [0.87-0.92] with I^2^ = 89.91%, while the pooled AUC value of the combined group was 0.81 [0.77-0.84] with I^2^ = 41.54%. The validation group had a pooled AUC of 0.84 [0.81-0.87] without heterogenicity, whereas the validation-free group had high heterogenicity (I^2 ^= 91.65%, P=0.000). The machine learning group had a pooled AUC of 0.83 [0.80-0.86] with I^2^ = 82.28%.

**Conclusion:**

AI can be considered as a potential tool to detect histological PNETs grades. Sample diversity, lack of external validation, imaging modalities, inconsistent radiomics feature extraction across platforms, different modeling algorithms and software choices were sources of heterogeneity. Standardized imaging, transparent statistical methodologies for feature selection and model development are still needed in the future to achieve the transformation of radiomics results into clinical applications.

**Systematic Review Registration:**

https://www.crd.york.ac.uk/prospero/, identifier CRD42022341852.

## Introduction

Pancreatic neuroendocrine tumors (PNETs), which account for 3–5% of all pancreatic tumors, are a heterogeneous group of tumors derived from pluripotent stem cells of the neuroendocrine system (1–3). In the past 10 years, the incidence and prevalence of PNETs have steadily increased (4–6). Unlike malignant tumors, PNETs are heterogeneous: they range from indolent to aggressive (7, 8). The World Health Organization (WHO) histological grading system is used to evaluate the features of PNETs, and a treatment strategy is developed accordingly (9, 10). Therefore, accurate evaluation of the histological grade is crucial for patients with PNETs; non-invasive methods are helpful, especially for tumors that are difficult to biopsy.

The application of artificial intelligence (AI) to medicine is becoming more common; it is useful in areas such as radiology, pathology, genomics, and proteomics (11–14), with broad applications in disease diagnosis and treatment (15–18). Owing to developments in AI technology, radiomic analysis can now be used to predict PNETs grade, with promising results (19, 20). A study by Guo et al. (21), which included 37 patients with PNETs, showed that the portal enhancement ratio, arterial enhancement ratio, mean grey-level intensity, kurtosis, entropy, and uniformity were significant predictors of histological grade. Luo et al. (22) found that by using specific computed tomography (CT) images, the deep learning (DL) algorithm achieved a higher accuracy rate than radiologists (73.12% vs. 58.1%) from G3 to G1/G2. Despite promising results, other studies with different methodologies have produced different findings. Thus, quantitative analysis will be valuable for comparing study efficacy and assessing the overall predictive power of AI in detecting the histological grade for PNETs.

In this review, we aimed to systematically summarize the latest literature on AI histological grade prediction for PNETs. By performing a meta-analysis, we aimed to evaluate AI accuracy and provide evidence for its clinical application and role in decision making.

## Materials and methods

This combined systematic review and meta-analysis was based on the Preferred Reporting Items for Systematic reviews and Meta-Analyses (PRISMA) guidelines. The study was registered in the Prospective Register of Systematic Reviews (PROSPERO ID: CRD42022341852).

### Search strategy

Primary publications were extracted from multiple electronic databases (PubMed, MEDLINE, Cocorane and Web of Science) in September 2023 using radiomics/DL/machine learning (ML)/AI on CT/magnetic resonance imaging (MRI) examinations of PNETs grade. The search terms consisted of ML, AI, radiomics, or DL, along with PNETs grade. The detail of search string was as follows: (radiomics or machine learning or deep learning or artificial intelligence)and (PNETs or pancreatic neuroendocrine tumors). The reference lists of generated studies were then screened for eligibility.

### Study selection

Two researchers determined the eligibility of each article by title and abstract evaluation and removed the duplicates. Case reports, non-original investigations (e.g., editorials, letters, and reviews), and studies that did not focus on the topic of interest were excluded. Based on the “PICOS” principle, the following inclusion criteria were designed. 1) All studies about PNETs grading which trained the models using only histology (and not biopsy) as gold standard were selected; 2) All PNETs grading predictive models built by AI were included. 3) Compared with physicians or models obtained from clinical and traditional imaging characteristics; 4) The main research purposes of the included studies were to differentiate the grades of PNETs; 5) Research types: case-control studies, cohort studies, nested case-control studies, and case-cohort studies; 6) English language. Exclusion criteria were: 1) Only the influencing factors were analyzed and a complete risk model was not built; 2) guides, case reports and non-original investigations (e.g., editorials, letters, meta-analyses and reviews); 3) other than English and animal studies. Any disagreements were resolved by consensus arbitrated by a third author.

### Data extraction

Data extraction was performed independently by two reviewers, and any discrepancies were resolved by a third reviewer. The extracted data included first author, country, year of publication, study aim, study type, number of patients, sample size, validation, treatment, reference standard, imaging modality and features, methodology, model features and algorithm, software segmentation, and use of clinical information (e.g., age, tumor stage, and expression biomarkers). A detailed description of the true positive (TP), false positive (FP), true negative (TN), false negative (FN), sensitivity, and specificity were recorded. The AUC value of the validation group along with the 95% confidence interval (CI) or standard error (SE) of the model was also collected if reported.

### Quality assessment

All included studies were independently assessed using the radiomics quality score (RQS), for image acquisition, radiomics feature extraction, data modeling, model validation, and data sharing. Each of the sixteen items was scored within a range of -8–36. Subsequently, the score was converted to a percentage, where -8 to 0 was defined as 0% and 36 as 100% (23).

The methodological quality of the included studies was accessed by the Quality Assessment of Diagnostic Accuracy Studies 2 (QUADAS-2) criteria (24). Two reviewers independently performed data extraction and quality assessment. Disagreements between the two reviewers were discussed at a research meeting until a consensus was reached.

### Statistical analysis

Three software packages, Stata, version 12.0, MedCalc for Windows, version 16.4.3 (MedCalc Software, Ostend, Belgium), and RevMan, version 5.3.21 were used for statistical analysis. A bivariate meta-analysis model was employed to calculate the pooled sensitivity, specificity, positive likelihood ratio (PLR), negative likelihood ratio (NLR), and diagnostic odds ratio (DOR), respectively. The symmetric receiver operating characteristic (SROC) curve was generated. The I^2^ value was used to assess statistical heterogeneity and estimate the percentage of variability among the included studies. An I^2^ value >50% indicated substantial heterogeneity, and a random-effects model was used to analyze the differences within and between studies. In contrast, if the value was <50%, it signified less heterogeneity and a fixed-effects model was used (25). Meta-regression and subgroup analysis were conducted to explore the sources of heterogeneity. Moreover, the sensitivity analysis was also performed to evaluate the stability. Deeks’ funnel plot was used to examine publication bias. A p value less than 0.05 was considered significant. Fagan’s nomogram was employed to examine the post-test probability.

## Results

### Literature selection

**Figure 1:**
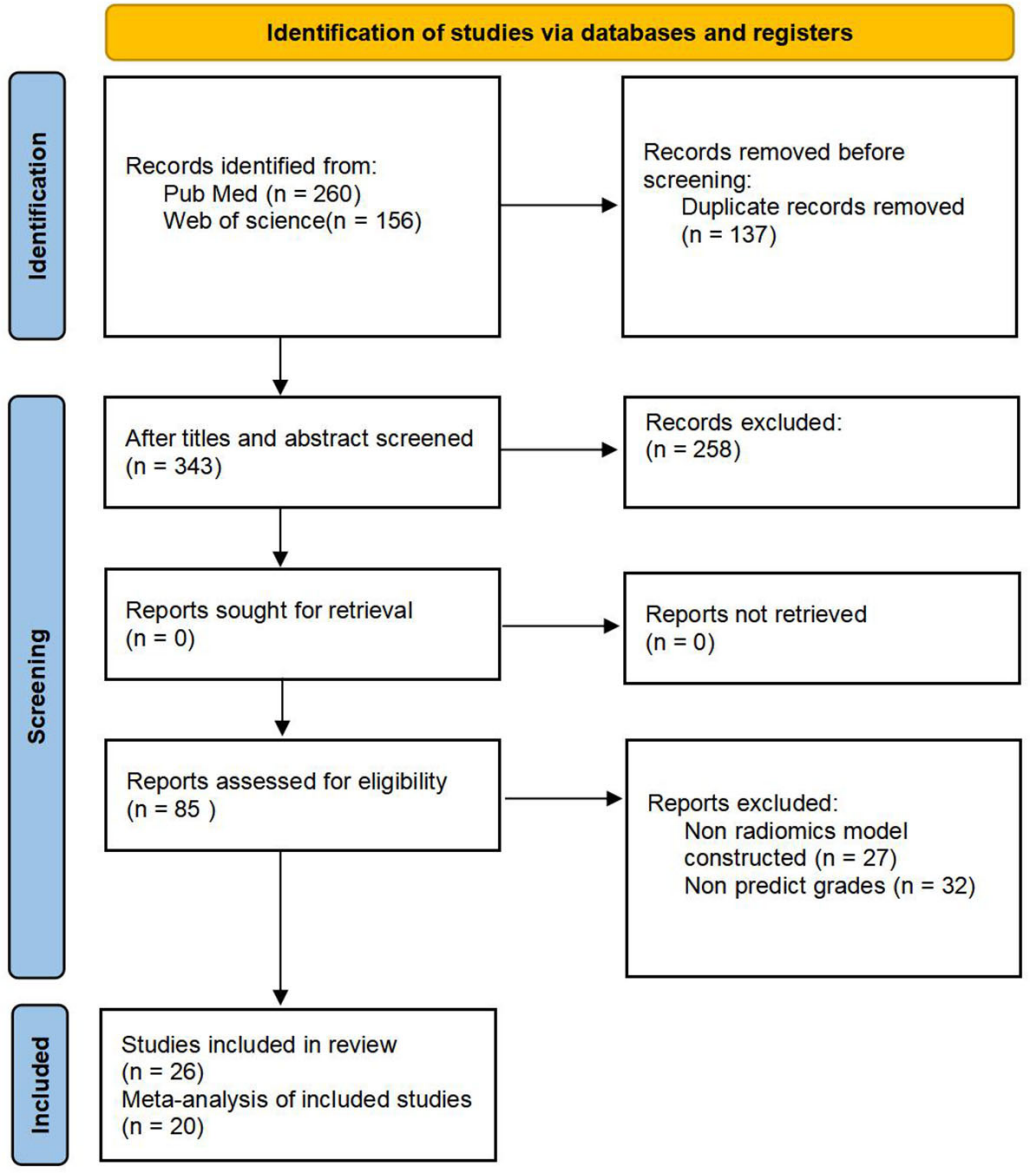
Flowchart of the article selection.

We retrieved 260 articles from PubMed and 156 from Web of Science; 137 were duplicates and were eliminated, resulting in 343 studies. After screening titles and abstracts, 85 potentially eligible articles were identified. After full-text review, six articles were excluded because of insufficient information; thus, 26 articles were included in this systematic review (21, 22, 26–49). Among them, six studies lacked information on positive and negative diagnosis values; therefore, only 20 articles were eligible for the meta-analysis. The results of the literature search are shown in **Figure 1**.

### Quality and risk bias assessment

As shown in **Table 1**, the selected articles were published between 2018 and 2023. The RQS average total and relative scores were 9.58 (2–20) and 26.60% (5.56–55.56%), respectively. No validation group in 13 studies, and five were based on two datasets from more than two distinct institutes. Due to the lack of prospective studies, deficiency of phantom studies on all scanners, absence of imaging at multiple time points, shortness of cost-effectiveness analysis, and unavailable open science and data, all the 11 included studies obtained the point of zero in these items. A detailed report of the RQS allocated by the expert reader is presented in **Supplementary Table S1**.

**Table 1:** Characteristic of all included studies.

Study	Aim	Imaging	Country	Year	Patient (N)	G1/G2/G3	Training set	Internal Validation set	External validation set	Data source	Reference standard	Algorithm architecture	Statistical methods	Total number of radiomics features included	Radiomic/ AI approach	Software Segmentation	Clinical model	Inclusion of Clinical Features in the Model	Clinical Features
Benedetti G(2021) (26)	G1 vs G2/G3	CT	Italy	2021	39	25/12/2	All	NR	NR	Single institution	pathology	NR	NR	69	ROI	NR;manual	Yes	No	NR
Bian Y(2020) (27)	G1 vs G2	CT	China	2020	102	52/50	All	NR	NR	Single institution	pathology	LASSO logistic regression	10-fold cross-validation	NR	ROI	3D Slicer;manual	No	Yes	age, sex, and body mass index, tumor size
Bian Y(2020-MRI) (28)	G1 vs G2/G3	MRI	China	2020	139	42/47/8(18/18/6)	96(42/47/8)	42(18/18/6)	NR	Single institution	pathology	LASSO logistic regression	10-fold cross-validation	14	ROI	3D Slicer;manual	Yes	Yes	tumor location, size, shape, margin, cystic changes, pancreatic and bile duct dilation, parenchymal atrophy, tumor intensity in T1WI and T2WI, the phase of peak enhanced value, enhanced mode, organ invasion, and vascular invasion.
Bian Y(2021-MRI) (29)	G1 vs G2/G3	MRI	China	2021	157	61/78/18	All	NR	NR	Single institution	pathology	LASSO logistic regression	10-fold cross-validation	7	ROI	3D Slicer;manual	No	Yes	age, sex, and BMI, T stage, N stage, clinical stage, perineural invasion, size, shape, phase of peak enhancement value, organs invasion, and vascular invasion
Canellas R(2018) (30)	G1 vs G2/G3	CT	USA	2018	101	63/35/3	All	NR	NR	Single institution	pathology	NR	NR	5	ROI	NR;manual	No	No	NR
Choi TW(2018) (31)	G1 vs G2/G3	CT	Korea	2018	66	45/16/5	All	NR	NR	Single institution	pathology	Logistic regression	NR	NR	ROI	NR;manual	No	No	NR
Gao X(2019) (32)	G1 vs G2 vs G3	MRI	China	2019	106	35/49/12(4/4/2)	96(35/49/12)	yes	10(4/4/2)	Two institutions	pathology	GAN;CNN	5-fold cross-validation	NR	ROI	Image J;manual	No	No	NR
Gu DS(2019) (33)	G1 vs G2/G3	CT	China	2019	138	57/69/12	104(38/66)	NR	34(19/15)	Two institutions	pathology	MRMR;RF	cross-validation	25	ROI	ITK-SNAP;manual	Yes	Yes	tumor margin
Guo C(2018) (21)	G1/G2 vs G3	CT	China	2018	37	13/11/13	All	NR	NR	Single institution	pathology	NR	NR	NR	ROI	Matlab;manual	Yes	Yes	NR
Guo C(2019) (34)	G1 vs G2 vs G3	MRI	China	2019	77	31/29/17	All	NR	NR	Single institution	pathology	NR	NR	NR	ROI	Omni-Kinetics software;manual	No	No	NR
Liu C(2022) (35)	G1 vs G2/G3	CT/MRI	China	2022	123	48/55/20	82(32/50)	41(16/25)	NR	Single institution	pathology	MRMR;LDA	NR	7	ROI	3D Slicer;manual	Yes	Yes	tumor size, phase of peak enhancement, enhanced mode, organs invasion, vascular invasion
Li W(2021) (36)	G1 vs G2	MRI	China	2021	48(51 lesions)	26/25	All	NR	NR	Single institution	pathology	Logistic regression	NR	NR	ROI	3D Slicer;manual	No	No	NR
Liang WJ(2019) (37)	G1 vs G2/G3	CT	China	2019	137	42/44;28/23	86(42/44)	NR	51(28/23)	Two institutions	pathology	LASSO logistic regression	10-fold cross-validation	8	ROI	3D Slicer;manual	Yes	Yes	the clinical stage and maximum diameter
Luo Y(2020) (22)	G1/G2 vs G3	CT	China	2020	112	NR	93	NR	19(13/6)	Two institutions	pathology	CNN;RF;SVM	8-fold cross-validation	NR	ROI	ITK-SNAP;manual	No	No	NR
Ohki K(2021) (38)	G1 vs G2/G3	CT/MRI	Japan	2020	32(33 lesions)	22/11	All	NR	NR	Single institution	pathology	NR	NR	7	ROI	syngo;manual	No	No	NR
Onofrio MD(2019) (39)	G1/G2 vs G3	CT	Italy	2018	100	31/52/17	All	NR	NR	Single institution	pathology	NR	NR	NR	ROI	3D sclicer;mannual	No	No	NR
Pulvirenti A(2021) (40)	G1 vs G2 vs G3	CT	USA	2021	150	94/47/9	105(66/33/6)	45(28/14/3)	NR	Single institution	pathology	SVM	NR	10	ROI	Scout Liver;manual	Yes	Yes	tumor shape, tumor margin,tumor attenuation on arterial phase, tumor uniformity, vascular invasion, CT value of tumor
Ricci C(2021) (41)	G1 vs G2/G3	CT	Italy	2021	68	29/30/2/7(unable to distinguish)	All	NR	NR	Single institution	pathology	LASSO logistic regression	cross-validation	NR	ROI	LifeX software;manual	Yes	Yes	symptoms, diameter at CT scan, enhancement homogenous vs heterogenous, margin well-defined vs ill-defined, TNM stage
Wang X(2022) (42)	G1 vs G2/G3	CT	China	2022	139	47/92	83(28/55)	56(19/37)	NR	Single institution	pathology	LASSO;SVM	5-fold cross-validation;the bootstrap	NR	ROI	NR;manual	No	Yes	T stage, dilated bile duct, clinical TNM stage, and tumor margin
Zhao ZR(2020) (43)	G1 vs G2	CT	China	2020	99	31/28(18/22)	59(31/28)	40(18/22)	NR	Single institution	pathology	SVM	5-fold cross-validation	6	ROI	3D sclicer;manual	No	No	NR
Zhou RQ(2019) (44)	G1 vs G2 vs G3	CT	China	2019	92	32/48/11	All	NR	NR	Single institution	pathology	Logistic Regression; SVM; LDA; Multilayer perceptron	leave-one-out cross-validation	NR	ROI	NR;manual	No	No	NR
Chiti G(2022) (45)	G1/G2 vs G3	CT	Italy	2022	78(GI:53;PNETs:25)	36/18/5/19(NEC)	58	20	NR	Single institution	pathology	LASSO logistic regression	NR	NR	ROI	3D sclicer;manual	No	No	NR
Mori M(2022) (46)	G1 vs G2/G3	CT	Italy	2022	101	76/25	70	31	NR	Single institution	pathology	MRMR	NR	NR	ROI	MIM software;manual	Yes	Yes	NR
Park YJ(2023) (47)	G1/G2 vs G3	[18F]FDG PET-CT	Japan	2023	58	7/26/25	47	11	NR	Single institution	pathology	Logistic regression;neural network;RF	5-fold cross validation;10-fold cross-validation	11	VOI	MIM software; semi-automatic	Yes	Yes	primary tumor size, hepatic metastasis, and extra-hepatic metastasis
Javed AA(2023) (48)	G1 vs G2/G3	CT	USA	2023	270	176/94(G2/G3)	201	69	NR	Single institution	pathology	RF	NR	10	ROI	3D sclicer;manual	No	No	NR
Zhu HB(2023) (49)	G1 vs G2/G3	MRI	China	2023	228	75/153	115	NR	113	Five centers	pathology	LASSO logistic regression	5-fold cross-validation	4	ROI	ITK-SNAP;manual	Yes	Yes	boundary, vascular invasion

LASSO,Least absolute shrinkage and selection operator; CNN, Convolutional Neural Network; RF, Random Forest; SVM, Support Vector Machine; GAN, Generative Adversarial Networks; MRMR, Minimum Redundancy Maximum Relevance; LDA, Linear Discriminant Analysis; NR, Not Reported.

Study quality and risk of bias were assessed using the QUADAS-2 criteria; the details are presented in **Supplementary Figure S1**. A majority of studies showed a low or unclear risk of bias in each domain. In the Patient Selection domain, one study is at high risk, 25 studies are at moderate risk, and this risk mainly arises from “discontinuous patient inclusion”. In the Index Test domain, 9 studies are at moderate risk due to the insufficient information provided to make a judgment, while others were at low risk. In the Reference Standard domain, only one study is at high risk because some patients cannot be accurately categorized to the specific grading in this study. In the Flow and Timing domain, all were at low risk.

### Publication bias

**Figure 2:**
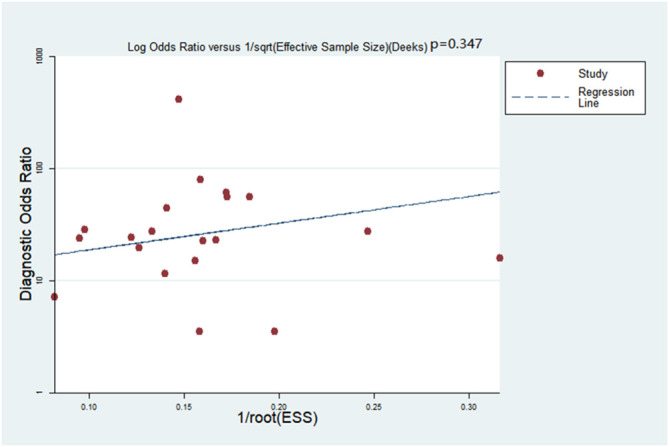
Deeks’ funnel plot evaluating the potential publication bias (p=0.034).

Deeks’ funnel plot asymmetry test was adopted to detect publication bias: no bias was detected within the meta-analysis (p=0.347, **Figure 2**).

Clinical diagnostic value of grading PNETs

**Figure 3:**
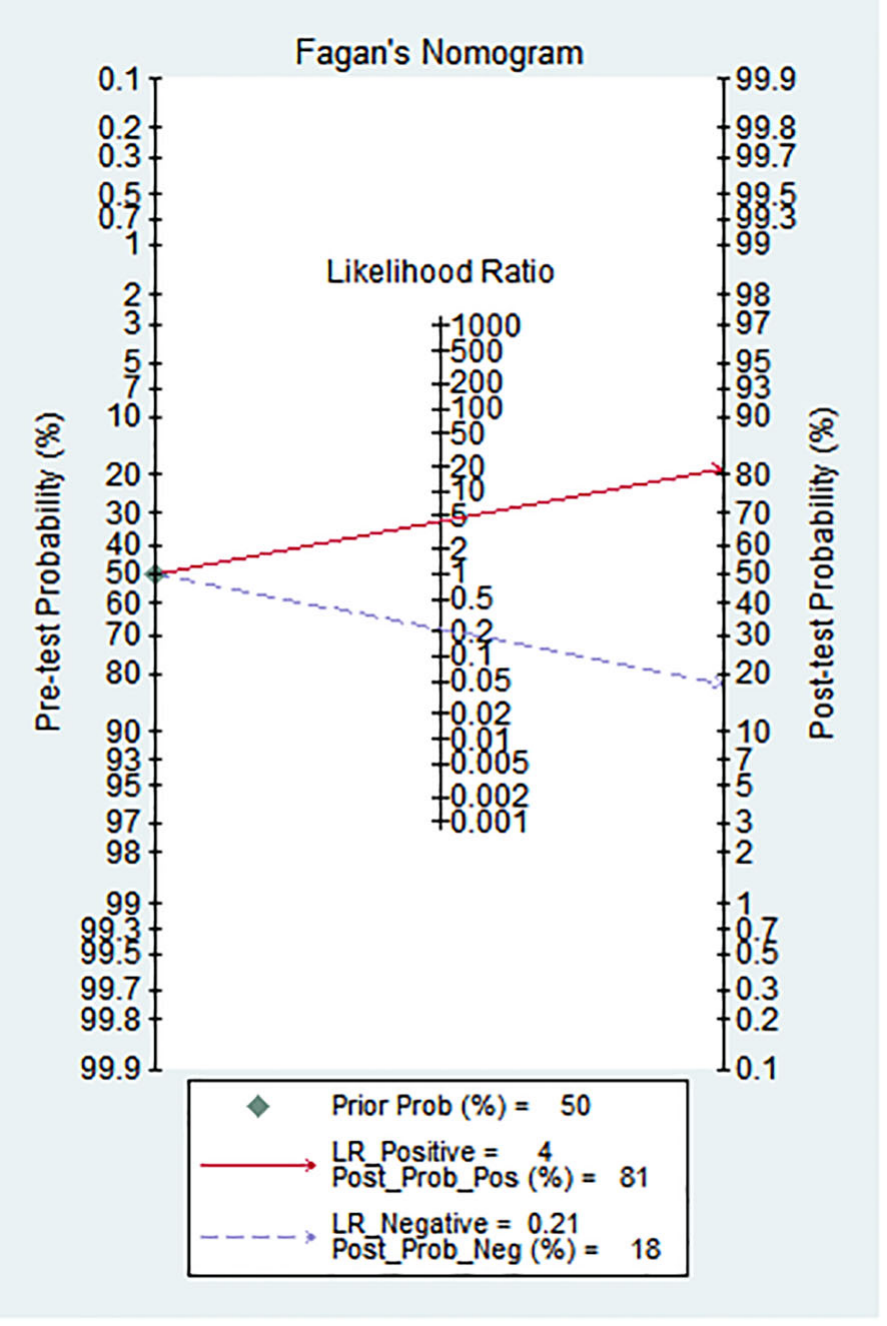
Fagan’s nomogram assessing the clinical diagnostic value of PNETs.

As shown in **Figure 3**, Fagan’s nomogram was useful for evaluating the diagnostic value of PNETs grade, and clinical application. The results showed an increase of post-test probability of the positive result (at 50%) to 81%, and a decrease of the negative result to 4%.

### Study characteristics

Study characteristics are summarized in **Table 1**. All studies employed a retrospective design, were published between 2018 and 2023, and the number of included patients was 32–270. Among the 26 studies, China was the main publication country (15 studies), followed by Italy (5 studies), the USA (3 studies), Korea (2 study), and Japan (1 study). Nineteen studies were based on CT and eight on MRI images, while two combined images from CT and MRI, and one applied for PET-CT. Thirteen of the 26 studies had validation sets; five were externally validated using data from another institute. The majority (20/26) used different kinds of ML classifications (such as Randon Rorest (RF); Support Vector Machine (SVM); Least absolute shrinkage and selection operator (LASSO) logistic regression), and two of them adopted Convolutional Neural Network (CNN). About half of the included studies (11/21) used models combined with clinical features (such as tumor size, tumor margin, TMN stage, etc.), while others used only radiomics features. Thirteen studies applied cross-validation to select stable features between observers.

The details of TP/FP/FN/TN and the models’ sensitivity and specificity are shown in **Table 2**. The highest area under the curve (AUC) value of the AI-based validation model was 0.99 (95% CI: 0.97–1.00). Six studies offered no details regarding TP/FP/FN/TN, and the AUC value of four studies was incomplete; thus, all of these six studies were excluded in meta-analysis.

**Table 2:** Results for accuracy to predict grade of PNETs.

Study	TP	FP	FN	TN	SE	SPE	AUC	SE	95%CI
Benedetti G(2021) (26)	16	1	9	13	0.64[0.43-0.82]	0.93[0.66-1.00]	0.80	0.08	0.71-1.00
Bian Y(2020) (27)	49	19	3	33	0.94[0.84-0.99]	0.63[0.49-0.76]	0.86	0.04	0.84-0.85
Bian Y(2020-MRI) (28)	15	6	3	18	0.83[0.59-0.96]	0.75[0.53-0.90]	0.74	0.09	0.52-0.88
Bian Y(2021-MRI) (29)	39	19	22	77	0.64[0.51-0.76]	0.80[0.71-0.88]	0.78	0.04	0.70-0.85
Canellas R(2018) (30)	NA	NA	NA	NA	NA	NA	0.65	NA	NA
Choi TW(2018) (31)	NA	NA	NA	NA	NA	NA	0.774	NA	NA
Gao X(2019) (32)	NA	NA	NA	NA	NA	NA	0.893	0.007	0.886-0.912
Gu DS(2019) (33)	13	2	2	17	0.87[0.60-0.98]	0.89[0.67-0.99]	0.90	0.05	0.80-1.00
Guo C(2018) (21)	22	2	2	11	0.92[0.73-0.99]	0.85[0.55-0.98]	0.96	0.05	0.77-0.97
Guo C(2019) (34)	29	0	2	17	0.94[0.79-0.99]	1.00[0.80-1.00]	0.99	0.01	0.97-1.00
Liu C(2022) (35)	13	4	3	21	0.81[0.54-0.96]	0.83[0.36-1.00]	0.85	0.06	0.71-0.94
Li W(2021) (36)	13	2	13	23	0.50[0.30-0.70]	0.92[0.74-0.99]	0.70	0.08	0.54-0.85
Liang WJ(2019) (37)	NA	NA	NA	NA	NA	NA	0.891	0.028	0.772-0.961
Luo Y(2020) (22)	11	1	2	5	0.85[0.55-0.98]	0.83[0.36-1.00]	0.81	0.04	0.71-0.88
Ohki K(2021) (38)	21	3	1	8	0.95[0.77-1.00]	0.73[0.39-0.94]	0.86	0.01	0.86-0.90
Onofrio MD(2019) (39)	14	12	3	71	0.82[0.57-0.96]	0.86[0.76-0.92]	0.92	0.04	0.78-0.92
Pulvirenti A(2021) (40)	7	6	8	24	0.47[0.21-0.73]	0.80[0.61-0.92]	NA	NA	NA
Ricci C(2021) (41)	25	8	4	31	0.86[0.68-0.96]	0.79[0.64-0.91]	0.91	0.00	0.90-0.92
Wang X(2022) (42)	31	2	6	17	0.84[0.68-0.94]	0.89[0.67-0.99]	0.88	0.01	0.87-0.89
Zhao ZR(2020) (43)	16	2	2	20	0.89[0.65-0.99]	0.91[0.71-0.99]	0.88	0.07	0.70-0.96
Zhou RQ(2019) (44)	NA	NA	NA	NA	NA	NA	0.85	NA	NA
Chiti G(2022) (45)	NA	NA	NA	NA	NA	NA	0.82	0.09	0.62-1.00
Mori M(2022) (46)	6	8	3	14	0.67[0.30-0.93]	0.64[0.41-0.83]	0.72	0.07	0.59-0.85
Park YJ(2023) (47)	4	1	1	4	0.80[0.28-0.99]	0.80[0.28-0.99]	0.83	0.04	0.73-0.93
Javed AA(2023) (48)	39	6	6	18	0.87[0.73-0.95]	0.75[0.53-0.90]	0.80	0.01	0.70-0.90
Zhu HB(2023) (49)	57	11	8	37	0.88[0.77-0.95]	0.77[0.63-0.88]	0.86	0.06	0.79–0.94

TP, true positive; FP, false positive; TN, true negative; FN, false negative; SE, sensitivity; SPE, specificity; AUC, area under the curve; SE, standard error; 95%CI, 95% confidence interval; NA, not applied.

## Meta-analysis

### Overall performance of the AI models

**Figure 4:**
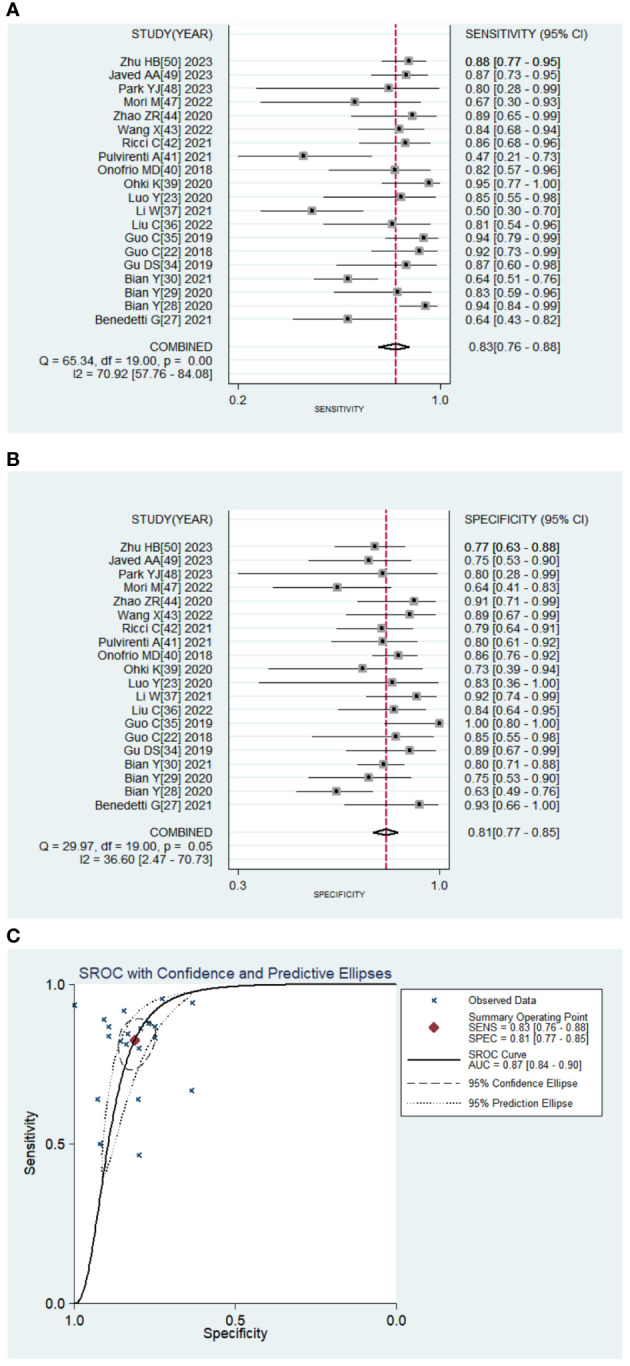
Pooled diagnostic accuracy of PNETs. **(A, B)** Forest plots of sensitivity, specificity; **(C)**. Summary receiver operator characteristic curve.

Twenty studies with 2639 patients were included in the meta-analysis, which provided data on TP/FP/FN/TN and model sensitivity and specificity, and 19 studies offered the AUC with 95% CI of the models. The results are reported in **Tables 2** and **3** and **Figure 4**. The AI models for PNETs showed an overall pooled sensitivity of 0.826 [0.759, 0.877], a pooled specificity of 0.812 [0.765, 0.851] and the pooled PLR and NLR were 4.382 [3.509, 5.472] and 0.215 [0.155, 0.298], respectively. Moreover, the pooled DOR was 20.387 [13.108, 31.706], and the AUC of the SROC curve was 0.89 [0.84-0.90] with I^2 ^= 90.42% [81.10-99.73], P=0.000.

**Table 3:** Subgroup analysis and estimates pooled of PNETs.

Group	models	AUROC	I²(%)	P value	Sensitivity	Specificity	Positive Likelihood Ratio	Negative Likelihood Ratio	Diagnostic Odds Ratio
All AI-based model	20	0.89 [0.84 - 0.90]	90.42 [81.10-99.73]	0.000	0.826 [0.759, 0.877]	0.812 [0.765, 0.851]	4.382 [3.509, 5.472]	0.215 [0.155, 0.298]	20.387 [13.108, 31.706]
CT	16	0.88 [0.85 - 0.91]	79.25 [55.2-100.0]	0.004	0.849 [0.786, 0.895]	0.803 [0.748, 0.847]	4.297 [3.386, 5.451]	0.189 [0.134, 0.266]	22.769 [14.707, 35.250]
MRI	6	0.83 [0.79 - 0.86]	71.55 [36.80-100.0]	0.015	0.791 [0.643, 0.888]	0.820 [0.764, 0.866]	4.407 [3.206, 6.058]	0.255 [0.141, 0.459]	17.304 [7.713, 38.822]
ML	15	0.84 [0.81 - 0.87]	89.88[79.90-99.86]	0.000	0.806 [0.727, 0.867]	0.789 [0.742, 0.829]	3.813 [3.156, 4.606]	0.246 [0.175, 0.346]	15.508 [10.196, 23.589]
non-ML	5	0.89 [0.86 - 0.92]	28.80[0.00-100.00]	0.124	0.879 [0.745, 0.947]	0.869 [0.799, 0.916]	6.686 [4.305, 10.383]	0.140 [0.063, 0.308]	47.863 [17.964, 127.526]
cross-validation	10	0.87 [0.83 - 0.91]	78.98[54.2-100.0]	0.004	0.831 [0.784, 0.871]	0.785 [0.737, 0.828]	3.523 [2.812, 4.414]	0.196 [0.127, 0.302]	20.262 [12.084, 33.973]
without cross-validation	10	0.88 [0.84 - 0.90]	75.30[45.77-100.0]	0.009	0.799 [0.670, 0.866]	0.835 [0.772, 0.884]	4.849 [3.365, 6.698]	0.241 [0.141, 0.413]	20.120 [9.171, 44.140]
clinical features included	10	0.81 [0.77 - 0.84]	41.54[0.00-100.0]	0.090	0.801 [0.707, 0.870]	0.795 [0.739, 0.842]	3.906 [2.983, 5.115]	0.251 [0.166, 0.379]	16.581 [9.466, 29.044]
only radiomics features	10	0.90 [0.87 - 0.92]	89.91[79.76-99.87]	0.000	0.847 [0.747, 0.913]	0.829 [0.749, 0.888]	4.970 [3.349, 7.377]	0.184 [0.109, 0.310]	27.034 [13.412, 54.492]
validation set	11	0.84 [0.81 - 0.87]	0.00[0.00-100.00]	0.375	0.823 [0.754, 0.876]	0.799 [0.744, 0.846]	4.106 [3.128, 5.389]	0.221 [0.155, 0.315]	15.574 [8.579, 28.273]
without validation set	9	0.89 [0.86 - 0.91]	91.65 [83.81-99.48]	0.000	0.836 [0.708, 0.914]	0.824 [0.741, 0.884]	4.741 [3.248, 6.920]	0.199 [0.110, 0.361]	23.766 [11.504, 49.095]
N>100	12	0.84 [0.81 - 0.87]	83.28 [64.73-100.0]	0.001	0.815 [0.737, 0.874]	0.784 [0.735, 0.826]	3.769 [3.086, 4.603]	0.236 [0.165, 0.337]	15.974 [10.228, 24.948]
N ≤ 100	8	0.91 [0.88 - 0.93]	77.39 [50.76-100.0]	0.006	0.847 [0.715, 0.925]	0.871 [0.799, 0.920]	6.560 [4.224, 10.187]	0.175 [0.091, 0.338]	37.404 [16.542, 84.577]

AUC, area under the curve; ML, machine learning.

Subgroup analysis based on the image source and AI methodology

Meta-regression was conducted and found there was no significant differences between groups (**Supplementary Table S2**). Then subgroup analysis was performed to compare studies evaluating the performance of different image sources: CT and MRI. Two models used both CT and MRI images; thus, 16 models extracted radiomic features from CT images and six models from MRI. The pooled SE, SP, PLR, and NLR were 0.849 [0.786, 0.895], 0.803 [0.748, 0.847], 4.297 [3.386, 5.451], and 0.189 [0.134, 0.266], respectively for CT models, and 0.791 [0.643, 0.888], 0.820 [0.764, 0.866], 4.407 [3.206, 6.058], 0.255 [0.141, 0.459], respectively for MRI models. The pooled DOR was 22.769 [14.707, 35.250] and 17.304 [7.713, 38.822] for CT and MRI models, respectively. The AUC of the SROC curve was 0.88 [0.85-0.91] with heterogeneity (I^2 ^= 79.25% [55.20-100.00], P=0.004) on CT images compared with MRI (AUC=0.83 [0.79-0.86], I^2 ^= 71.55%[36.80-100.00], P=0.015).

Subgroup analysis of different AI methodologies was used to compare algorithm architecture; most models not only applied ML classifiers, but more than one classifier. In total, 15 models were conducted using ML for PNETs. The pooled SE, SP, PLR, and NLR were 0.806 [0.727, 0.867, 0.789 [0.742, 0.829], 3.813 [3.156, 4.606], and 0.246 [0.175, 0.346], respectively. The pooled DOR was 15.508 [10.196, 23.589] and the AUC of the SROC curve was 0.84[0.81-0.87] with heterogenicity, I^2 ^= 89.88%[79.90-99.86]. Of the remaining three models for non-ML, the pooled AUC value was 0.89 [0.86-0.92] with I^2 ^= 28.80[0.00-100.00] (**Table 3**).

There were ten models using cross-validation to select the best features and models. The group with cross-validation had a pooled AUC of 0.87 [0.83-0.91] with I^2 ^= 78.98%, while the group without was 0.88 [0.84-0.90] with I^2 ^= 75.30%. The pooled SE, SP, PLR, and NLR were 0.831 [0.784, 0.871], 0.785 [0.737, 0.828], 3.523 [2.812, 4.414] and 0.196 [0.127, 0.302], respectively for the cross-validation group, and 0.799 [0.670, 0.866], 0.835 [0.772, 0.884], 4.849 [3.365, 6.698], and 0.241 [0.141, 0.413], respectively for the group without ICC. The pooled DOR were 20.262 [12.084, 33.973] and 20.120 [9.171, 44.140] for the groups with and without ICC, respectively.

### Subgroup analysis based on dataset characteristics

We also compared the models that included clinical data and by utilizing radiomics features only, and found that clinical features reduced heterogenicity. The pooled SE, SP, PLR, and NLR were 0.801 [0.707, 0.870], 0.795 [0.739, 0.842], 3.906 [2.983, 5.115], and 0.251 [0.166, 0.379], respectively for the group including clinical data, and 0.847 [0.747, 0.913], 0.829 [0.749, 0.888], 4.970 [3.349, 7.377], and 0.184 [0.109, 0.310], respectively for the radiomics-only group. The pooled DOR for the radiomics group was 27.034 [13.412, 54.492], and the AUC of the SROC curve was 0.81 [0.77-0.84] with I^2 ^= 41.54%, which was a little higher than that of the included clinical data group (DOR: 16.581 [9.466, 29.044]); AUC: 0.90 [0.87-0.92]) (**Table 3**).

Moreover, 11 models were validated, while nine models were not. The pooled SE, SP, PLR, and NLR were 0.823 [0.754, 0.876], 0.799 [0.744, 0.846], 4.106 [3.128, 5.389], and 0.221 [0.155, 0.315], respectively for the validated group, and 0.836 [0.708, 0.914], 0.824 [0.741, 0.884], 4.741 [3.248, 6.920], and 0.199 [0.110,0.361], respectively for the control group. The pooled DOR for the validated group was 15.574 [8.579, 28.273] and 23.766 [11.504, 49.095] for the control group. The AUC of the SROC curve was 0.84 [0.81-0.87] without heterogeneity for the validation group and 0.89 [0.86-0.91] with I^2 ^= 91.65% for the control group.

In a subgroup analysis based on the number of patients, the pooled results of 12 models, which included >100 patients, were 0.815 [0.737, 0.874], 0.784 [0.735, 0.826], 3.769 [3.086, 4.603], and 0.236 [0.165, 0.337] for the pooled SE, SP, PLR, and NLR, respectively. For the remaining eight models, the pooled SE, SP, PLR, and NLR were 0.847 [0.715, 0.925], 0.871 [0.799, 0.920], 6.560 [4.224, 10.187], and 0.175 [0.091, 0.338], respectively. The pooled DOR and the AUC values for the two groups were 15.974 [10.228, 24.948] and 0.84 [0.81-0.87] vs. 37.404 [16.542, 84.577] and 0.91 [0.88-0.93] (**Table 3**).

## Discussion

PNETs are a heterogeneous group of malignancies: they can be grouped into grades G1, G2, and G3 according to mitotic count and Ki-67 index (1–3). Accurate classification of PNETs grades is important for treatment selection, prognosis, and follow-up. However, due to the heterogeneity of PNETs, tumor grading may not be uniform within a single lesion or between different lesions in the same patient (7, 8). Moreover, histology is currently the only validated tool to grade tumors and describe their characteristics; surgery and endoscopic biopsy are used clinically to analyze the histological grade of PNETs. However, it is difficult to perform a satisfactory biopsy for PNETs located around major vessels, or small tumors—especially using fine-needle aspiration biopsy (50–53). Therefore, the detection of histological grades based on radiological images is also an important diagnostic tool. With increasing AI application in medical fields, we believe that AI-based models can enhance the prediction value of tumor grading. To the best of our knowledge, we are only aware of few and insufficiently updated systematic review on this topic that has evaluated the diagnostic accuracy of radiomics.

In our study, we investigated the ability of imaging-based AI to detect PNETs histologic grading. Our results showed that AI-based grading of PNETs with an AUC of 0.89 [0.84 - 0.90] exhibited good performance but high heterogeneity (I^2 ^= 90.42% [81.10-99.73], P = 0.000). Among the included studies, we found considerable heterogeneity in pooled sensitivity and specificity. Moreover, according to our sensitive analysis, 3 articles (29, 40, 46) had poor robustness and may be one of the sources of heterogeneity (**Figure S2**). There was no significant publication bias between studies.

The diagnostic performance of the radiomics model varied with the strategies employed. CT and MRI images are the main sources for analyzing PNETs. Because of its high availability and low cost, CT is widely used than MRI. In this study, we found that imaging techniques may be influencing factors of prediction power, but not independently so. CT was more commonly used (16 studies) and showed better performance than MRI (6 studies) in grading PNETs, with an AUC of 0.88 [0.85-0.91] vs. 0.83 [0.79-0.86]. Although unconfirmed, we speculate that CT may be more powerful for obtaining vessel enhancement characteristics and observing the neo-vascular distribution, which is useful in vascularly-rich PNETs (54). Future studies are needed to validate this finding. We had only one study applied PET-CT grading PNETs and found AUROC of 0.864 in the tumor grade prediction model which showed good forecasting ability (47). Thus, more investigation into PET-CT will be useful in developing AI models, which showing good predictive performance (AUC = 0.992) and can detect cell surface expression of somatostatin receptors (55, 56).

Clinical data such as age, gender, tumor size, tumor shape, tumor margin and CT stage are closely related to the pathogenic process of PNETs and therefore should not be ignored in diagnostic models (27–29, 47, 49).,Liang et al. (37) built a combined model which can improve the performance (0.856, [0.730–0.939] vs. 0.885 [0.765–0.957]). Wang et al. (42) found that the addition of clinical features can improve the radiomics models (from 0.837 [0.827–0.847] to 0.879 [0.869–0.889]). However, we found that including clinical factors did not always result in better performance but did decrease the heterogenicity (AUC of 0.81 [0.77-0.84] with I^2 ^= 41.94% vs. 0.90 [0.87-0.92] with I^2 ^= 89.91%). This may due to the data are processed differently, such as age or other clinical numerical data can be easily quantified by radiomic modeling (i.e., age as a variable in an algorithm or function). And in clinical models, age regarded as risk factors always varied in different situations. Therefore, future radiomics analyses should incorporate clinical features to create more reliable models or add radiomics features to existing diagnostic models to validate their true diagnostic power.

The lack of standardized quality control and reporting throughout the workflow limits the application of radiomics (17, 57). For example, validation/testing data must remain completely independent or hidden until validation/testing is performed in order to create generalizable predictive models at each step of a radiomics study. In our study, 11 studies of 20 had validation set and only 3 had external validation. Lack of proper external validation would influence the transportability and generalizability of the models in the studies and also hamper the conclusions of the review. Moreover, according to our findings, lacking validation sets was also one of the main causes of heterogeneity. There should be no direct comparison between the results obtained by studying only the primary cohort and those obtained by studying both the primary and validation cohorts. Validated models should be considered more reliable and promising, even if the reported performance is lower.

As shown in **Table 1**, there were also a wide variety of feature extraction and model selection methods, and although AI classifiers did not show outstanding diagnostic performance in our evaluation, it is undeniably a future research direction and trend. Most of the included studies used more than one machine learning or deep learning for feature selection or classification, but the best performing AI classifiers varied from study to study. To date, there are no universal and well-recognized classifiers, and the characteristics of the samples are a key factor affecting the performance of classifiers (58, 59). Finding uniform and robust classifiers for specific medical problems has always been a challenge.

Despite the encouraging results of this meta-analysis, the overall methodological quality of the included literature was poor, reducing the reliability and reproducibility of radiomics models for clinical applications. Lack of prospective studies with scanner modeling studies, lack of imaging studies at multiple time points, insufficient validation and calibration validity of the models, short time frame for cost-effectiveness analyses, insufficient cost-effectiveness analyses, and lack of publicly available science and data contributed to the low RQS scores. In addition, only half of the studies were internally validated and less independent external validation. To further standardize the process and improve the quality of radiomics, the RQS should be used not only to assess the methodological quality of radiomics studies, but also to guide the design of radiomics studies (17).

Diversity of the samples, inconsistencies with radiomics feature extraction across platforms, different modeling algorithms, and simultaneous incorporation of clinical features may all account for the high heterogenicity of the combined models. According to our sub-analysis results, the heterogenicity mainly came from the different imaging materials (CT vs MRI), the algorithm architecture (ML vs non-ML), whether validated or not and clinical features included. Thus, standardized imaging, a standardized independent and robust set of features, as well as validation even external validation are all approaches to lower the heterogenicity and highlights for attention in future research. To sum, the AI method was effective in the preoperative prediction of PNETs grade; this may help with the understanding of tumor behavior, and facilitate vision-making in clinical practice.

Our study has several limitations. First, most included studies were single-center and retrospective, inevitably causing patient selection bias. Second, different methods were investigated, including the type of imaging scans utilized, the type and number of radiological features studied, the choice of software, and the type of analysis/methods implemented, thus leading to the high heterogeneity among studies. Therefore, some pooled estimates of the quantitative results must be interpreted with caution. Further prospective studies could validate these results; a stable method of data extraction and analysis is important for developing a reproducible AI model.

## Conclusions

Overall, this meta-analysis demonstrated the value of AI models in predicting PNETs grading. According to our result, diversity of the samples, lack of external validation, imaging modalities, inconsistencies with radiomics feature extraction across platforms, different modeling algorithms and the choice of software all are sources of heterogeneity. Thus, standardized imaging, as well as a standardized, independent and robust set of features will be important for future application. Multi-center, large-sample, randomized clinical trials could be used to confirm the predictive power of image-based AI systems in clinical practice. To sum, AI can be considered as a potential tool to detect histological PNETs grades.

## Data availability statement

The original contributions presented in the study are included in the article/**Supplementary Material**. Further inquiries can be directed to the corresponding authors.

## Author contributions

QY: Conceptualization, Methodology, Visualization, Writing – original draft. YC: Data curation, Methodology, Software, Writing – original draft. CL: Data curation, Investigation, Software, Writing – original draft. HS: Data curation, Investigation, Validation, Writing – original draft. MH: Formal analysis, Software, Writing – original draft. ZW: Investigation, Validation, Writing – original draft. SH: Data curation, Formal analysis, Funding acquisition, Writing – review & editing. CZ: Funding acquisition, Project administration, Supervision, Writing – review & editing. BH: Funding acquisition, Project administration, Supervision, Writing – review & editing.

## Supplementary material

Supplementary Figure 1 The quality assessment of 26 included studies by QUADAS-2 tool.

Supplementary Figure 2 The sensitive analysis of 26 included studies.
