# Supplementary figures and images for: Predicting histologic grades for pancreatic neuroendocrine tumors by radiologic image-based artificial intelligence: a systematic review and meta-analysis
Source: Front Oncol. 2024 Apr 23;14:1332387. doi: 10.3389/fonc.2024.1332387 (PMC11080013; doi:10.3389/fonc.2024.1332387)

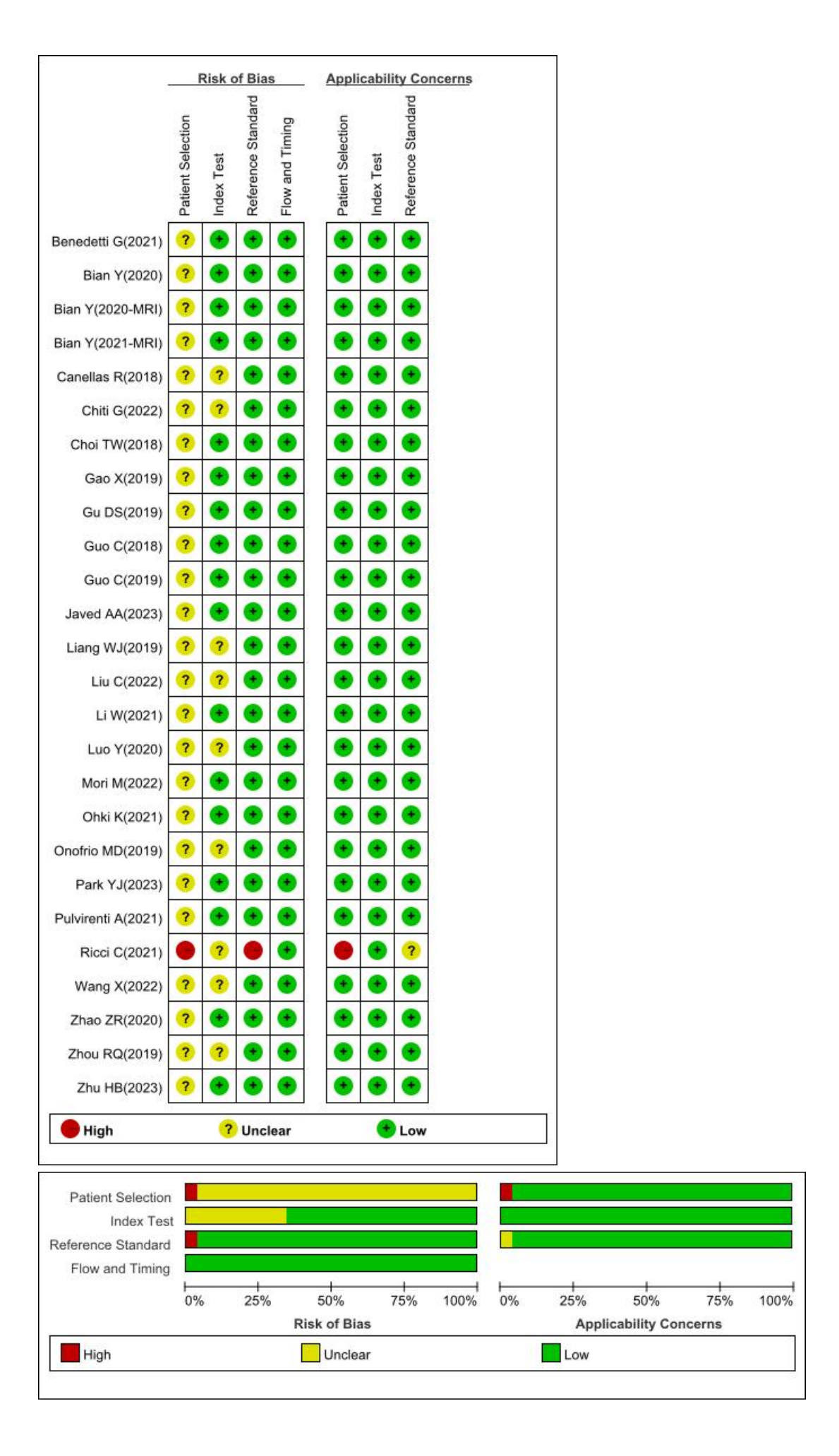

Supplement: Supplementary Figure 1 — The quality assessment of 26 included studies by QUADAS-2 tool. [file Image_1.png]

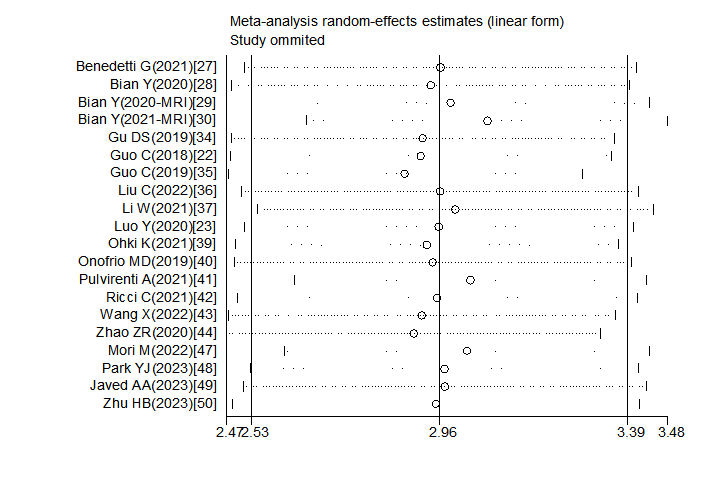

Supplement: Supplementary Figure 2 — The sensitive analysis of 26 included studies. [file Image_2.tif]
