# Supplementary material for: Predicting histologic grades for pancreatic neuroendocrine tumors by radiologic image-based artificial intelligence: a systematic review and meta-analysis
Source: Front Oncol. 2024 Apr 23;14:1332387. doi: 10.3389/fonc.2024.1332387 (PMC11080013; doi:10.3389/fonc.2024.1332387)
Supplement: Supplementary file 3 [file Table_1.docx]

Table S1. RQS score of all included studies.

| **No** | **RQS scoring item** | Benedetti G(2021)[27] | Bian Y(2020)[28] | Bian Y(2020-MRI)[29] | Bian Y(2021-MRI)[30] | Canellas R(2018)[31] | Choi TW(2018)[32] | Gao X(2019)[33] | Gu DS(2019)[34] | Guo C(2018)[22] | Guo C(2019)[35] | Liu C(2022)[36] | Li W(2021)[37] | Liang WJ(2019)[38] | Luo Y(2020)[23] | Ohki K(2021)[39] | Onofrio MD(2019)[40] | Pulvirenti A(2021)[41] | Ricci C(2021)[42] | Wang X(2022)[43] | Zhao ZR(2020)[44] | Zhou RQ(2019)[45] | Chiti G(2022)[46] | Mori M(2022)[47] | Park YJ(2023)[48] | Javed AA(2023)[49] | Zhu HB(2023)[50] |
| --- | --- | --- | --- | --- | --- | --- | --- | --- | --- | --- | --- | --- | --- | --- | --- | --- | --- | --- | --- | --- | --- | --- | --- | --- | --- | --- | --- |
| 1 | Image protocol quality | 1 | 1 | 1 | 1 | 1 | 1 | 1 | 1 | 1 | 1 | 1 | 1 | 1 | 1 | 1 | 0 | 0 | 0 | 1 | 1 | 1 | 0 | 1 | 1 | 1 | 1 |
| 2 | Multiple segmentations | 1 | 1 | 1 | 1 | 1 | 1 | 1 | 1 | 1 | 1 | 1 | 1 | 1 | 1 | 1 | 1 | 1 | 1 | 1 | 1 | 1 | 1 | 1 | 1 | 1 | 1 |
| 3 | Phantom study on all scanners | 0 | 0 | 0 | 0 | 0 | 0 | 0 | 0 | 0 | 0 | 0 | 0 | 0 | 0 | 0 | 0 | 0 | 0 | 0 | 0 | 0 | 0 | 0 | 0 | 0 | 0 |
| 4 | Imaging at multiple time points | 0 | 0 | 0 | 0 | 0 | 0 | 0 | 0 | 0 | 0 | 0 | 0 | 0 | 0 | 0 | 0 | 0 | 0 | 0 | 0 | 0 | 0 | 0 | 0 | 0 | 0 |
| 5 | Feature reduction or adjustment for multiple testing | 3 | 3 | 3 | 3 | 3 | 3 | 3 | 3 | 3 | 3 | 3 | 3 | 3 | 3 | 3 | 3 | 3 | 3 | 3 | 3 | 3 | 3 | -3 | 3 | 3 | 3 |
| 6 | Multivariable analysis with non-radiomics features | 0 | 1 | 0 | 0 | 0 | 1 | 0 | 1 | 0 | 0 | 0 | 0 | 0 | 0 | 0 | 0 | 0 | 1 | 0 | 0 | 0 | 0 | 0 | 1 | 0 | 1 |
| 7 | Detect and discuss biological correlates | 1 | 1 | 1 | 1 | 1 | 1 | 1 | 1 | 1 | 1 | 1 | 1 | 1 | 1 | 1 | 1 | 1 | 1 | 1 | 1 | 1 | 1 | 1 | 1 | 0 | 1 |
| 8 | Cut-off analyses | 1 | 0 | 0 | 1 | 1 | 1 | 0 | 0 | 0 | 0 | 0 | 0 | 0 | 0 | 1 | 1 | 0 | 0 | 0 | 0 | 1 | 0 | 0 | 1 | 0 | 1 |
| 9 | Discrimination statistics | 2 | 2 | 2 | 2 | 0 | 0 | 2 | 2 | 2 | 2 | 2 | 2 | 1 | 2 | 2 | 2 | 2 | 2 | 2 | 2 | 2 | 0 | 0 | 0 | 0 | 2 |
| 10 | Calibration statistics | 0 | 0 | 2 | 0 | 0 | 0 | 0 | 2 | 0 | 0 | 2 | 2 | 0 | 0 | 0 | 0 | 0 | 0 | 2 | 2 | 0 | 0 | 0 | 0 | 0 | 2 |
| 11 | Prospective study registered in a trial database | 0 | 0 | 0 | 0 | 0 | 0 | 0 | 0 | 0 | 0 | 0 | 0 | 0 | 0 | 0 | 0 | 0 | 0 | 0 | 0 | 0 | 0 | 0 | 0 | 0 | 0 |
| 12 | Validation | -5 | -5 | 2 | -5 | -5 | -5 | 3 | 3 | -5 | -5 | 3 | 2 | -5 | 3 | -5 | -5 | 2 | -5 | 2 | 2 | -5 | -5 | 2 | 2 | 2 | 5 |
| 13 | Comparison to ‘gold standard’ | 2 | 2 | 2 | 2 | 2 | 2 | 2 | 2 | 2 | 2 | 2 | 2 | 2 | 2 | 2 | 2 | 2 | 2 | 2 | 2 | 2 | 2 | 2 | 2 | 2 | 2 |
| 14 | Potential clinical utility | 0 | 2 | 2 | 2 | 0 | 0 | 0 | 2 | 0 | 0 | 2 | 2 | 0 | 0 | 0 | 0 | 0 | 0 | 0 | 0 | 0 | 0 | 1 | 1 | 1 | 1 |
| 15 | Cost-effectiveness analysis | 0 | 0 | 0 | 0 | 0 | 0 | 0 | 0 | 0 | 0 | 0 | 0 | 0 | 0 | 0 | 0 | 0 | 0 | 0 | 0 | 0 | 0 | 0 | 0 | 0 | 0 |
| 16 | Open science and data | 0 | 0 | 0 | 0 | 0 | 0 | 0 | 0 | 0 | 0 | 0 | 0 | 0 | 0 | 0 | 0 | 0 | 0 | 0 | 0 | 0 | 0 | 0 | 0 | 0 | 0 |
|  | Total points: −8 to 0 = 0%, 36 = 100% | 6 | 8 | 16 | 8 | 4 | 5 | 13 | 18 | 5 | 5 | 17 | 16 | 4 | 13 | 6 | 5 | 11 | 5 | 14 | 14 | 6 | 2 | 5 | 13 | 10 | 20 |
|  | Mean score(%) | 16.67 | 22.22 | 44.44 | 22.22 | 11.11 | 13.89 | 36.11 | 50.00 | 13.89 | 13.89 | 47.22 | 44.44 | 11.11 | 36.11 | 16.67 | 13.89 | 30.56 | 13.89 | 38.89 | 38.89 | 16.67 | 5.56 | 13.89 | 36.11 | 27.78 | 55.56 |

RQS, radiomics quality score
