# Supplementary material for: Predicting histologic grades for pancreatic neuroendocrine tumors by radiologic image-based artificial intelligence: a systematic review and meta-analysis
Source: Front Oncol. 2024 Apr 23;14:1332387. doi: 10.3389/fonc.2024.1332387 (PMC11080013; doi:10.3389/fonc.2024.1332387)
Supplement: Supplementary file 4 [file Table_2.docx]

Table S2. Meta-regression of PNETs.

| Models | exp(b) | Std. Err. | P | 95%CI |
| --- | --- | --- | --- | --- |
| Imaging | 0.690 | 0.320 | 0.434 | 0.261-1.827 |
| ML | 0.350 | 0.197 | 0.079 | 0.108-1.143 |
| Cross validation | 0.800 | 0.366 | 0.632 | 0.306-2.092 |
| Clinical features included | 0.689 | 0.315 | 0.425 | 0.263-1.800 |
| Validation set | 1.286 | 0.581 | 0.585 | 0.497-3.324 |
| Number of patients | 0.445 | 0.211 | 0.105 | 0.164-1.204 |

ML,machine learning
